# The surgical management of fibrous dysplasia of bone

**DOI:** 10.1186/1750-1172-7-S1-S1

**Published:** 2012-05-24

**Authors:** Robert P Stanton, Ernesto Ippolito, Dempsey Springfield, Lynn Lindaman, Shlomo Wientroub, Arabella Leet

**Affiliations:** 1 Pediatric Orthopedics, Nemours Children’s Clinic, Pensacola, FL, USA; 2 Department of Orthopedic Surgery, University of Rome Tor Vergata, Rome, Italy; 3 Massachusetts General Hospital, Harvard University, Boston, MA, USA; 4 Lindaman Orthopedics Des Moines, IA, USA; 5 Dana Children’s Hospital, Tel Aviv University, Tel Aviv, Israel; 6 Johns Hopkins University, Baltimore, MD, USA

## Abstract

The surgical management of Polyostotic Fibrous Dysplasia (FD) of bone is technically demanding. The most effective methods to manage the associated bone deformity remain unclear. The marked variation in the degree and pattern of bone involvement has made it difficult to acquire data to guide the surgeon’s approach to these patients. In light of the paucity of data, but need for guidance, recognized experts in the management of these patients came together at the National Institutes of Health in Bethesda, Maryland as part of an International meeting to address issues related to fibrous dysplasia of bone to discuss and refine their recommendations regarding the surgical indications and preferred methods for the management of these challenging patients. The specific challenges, recommended approaches, and “lessons learned” are presented in hopes that surgeons faced with typical deformities can be guided in the surgical reconstruction of both children and adults with FD.

## The initial evaluation of an adult patient with fibrous dysplasia

In the majority of the patients with fibrous dysplasia (FD) in whom the diagnosed is made in adulthood, FD is an incidental finding. Typically a bone lesion is detected on radiographs that were performed to evaluate a common injury, such as a sprain. Occasionally, the adult patient may present with dull, aching pain and subsequent radiographs may detect a bone lesion. As a first step in the evaluation, a full-body ^99^Tc-methylene diphosphonate (MDP) bone scan is recommended to not only evaluate the biologic activity of the index lesion, but to detect any additional lesions that may exist throughout the skeleton. If the radiographic appearance is typical (thinning of the cortex without periosteal reaction with a matrix appearance that has been characterized as resembling “ground glass”), most often the diagnosis may be rendered without additional imaging studies (i.e. computed tomography (CT), or magnetic resonance imaging (MRI)). Biopsy is indicated for histologic confirmation only in cases that do not present a typical radiographic appearance.

### Management of adult monostotic disease

Treatment decisions for adult patients with monostotic disease depend entirely on the presence of symptoms. The typical lesion, which is identified incidentally and remains asymptomatic, should be treated with observation and serial radiographs at an interval determined by consensus between the patient and the surgeon until they are satisfied that the lesion is biologically inactive and mechanically insignificant. When surgical intervention is indicated, monostotic lesions are typically treated with conventional surgical procedures [1]. In the absence of clinical symptoms, typical monostotic disease may be observed without specific intervention. In select patients, surgical management may be indicated for a variety of reasons. In some patients, the fear of malignant disease may be so profound that the surgeon is unable to adequately reassure the patient of the benign nature of the process. In other patients, the lesion may cause a true mechanical deficit that has led to bone pain or fracture and therefore intervention may be indicated. Typical orthopedic procedures to remove the lesion and to graft the defect may be used in these cases. The use of internal fixation should be considered in most cases to aid in immediate weight bearing and to augment the strength of the bone. If recurrent FD results in the resorption of the graft, generally accepted principles of orthopedic tumor surgery are followed. In MFD in adults, one can expect low levels of tumor recurrence.

## The initial evaluation of a child with fibrous dysplasia

Typically a child with FD will consult the orthopedic surgeon for complaints of pain, limp, or management of a pathologic fracture through an area of FD. If the child also has café-au-lait macules, the diagnosis of McCune-Albright Syndrome (MAS) is easily made. The classic diagnostic criteria for MAS had been FD, café-au-lait macules, and precocious puberty [2,3], but better understanding of the molecular and developmental etiology of FD/MAS has led the acceptance of the fact that any combination of one or more of the typical features of MAS (FD, café-au-lait macules, and/or hyperfunctioning endocrinopathies such as gonadotropin-independent precocious puberty, hyperthyroidism, growth hormone excess, etc.) warrants the diagnosis of MAS. In fact, given that the molecular etiology of even monostotic FD (MFD) is the same activating mutation in G_s_α as is found in full spectrum MAS [4], MFD can be considered a *forme fruste* of MAS.

The consensus of the authors is that the initial evaluation of the child should begin with a ^99^Tc-MDP bone scan to assess for the presence and/or extent of polyostotic FD (PFD). Before age six, and especially before the age of three, the bone scan may not show all areas that will ultimately be involved with FD, as small foci of FD may not be detected by the bone scan. After age six, affected areas of FD are usually detectable, and the family can be reassured that it is very unlikely that any “new” areas of FD, and certainly no new areas of clinical significance beyond what is already seen, will subsequently appear [5]. However, parents should also be informed that affected areas identified at a young age may progress. All young patients diagnosed with FD, and especially those with PFD require an evaluation by an endocrinologist, even in the absence of history or clinical findings suggestive of endocrine dysfunction. In addition, older patients with MFD, who have any history or clinical findings suggestive of endocrine dysfunction, should be referred for an endocrine evaluation. Patients whose bone scan shows cranial or facial involvement will require an evaluation by a craniofacial specialist. While CT imaging is often necessary to evaluate craniofacial FD, the consensus of the authors is that CT and/or MRI evaluation of long bone and spinal lesions are rarely indicated. Biopsy and/or molecular diagnosis (gene testing for mutations in *GNAS*) is rarely indicated in polyostotic disease, as the diagnosis can be rendered confidently on the basis of the history, physical examination and radiographs. When surgical procedures are required material may be obtained for histologic and/or molecular diagnostic confirmation or research purposes.

## Management and follow-up of pediatric polyostotic fibrous dysplasia

**Figure 1:**
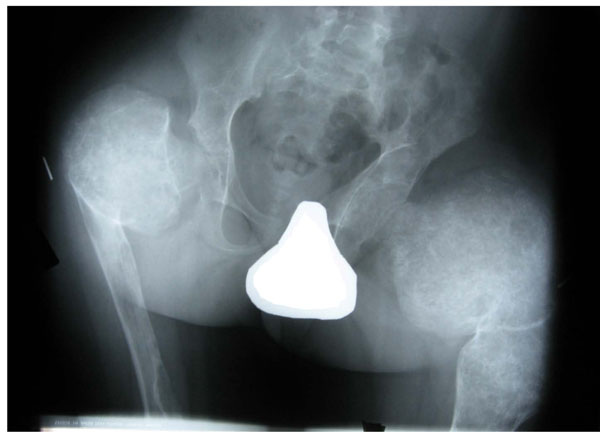
Fibrous dysplasia of the proximal femur. The radiograph demonstrates severe femoral involvement with deformity, and a typical ground glass appearance in both proximal femora.

**Figure 2:**
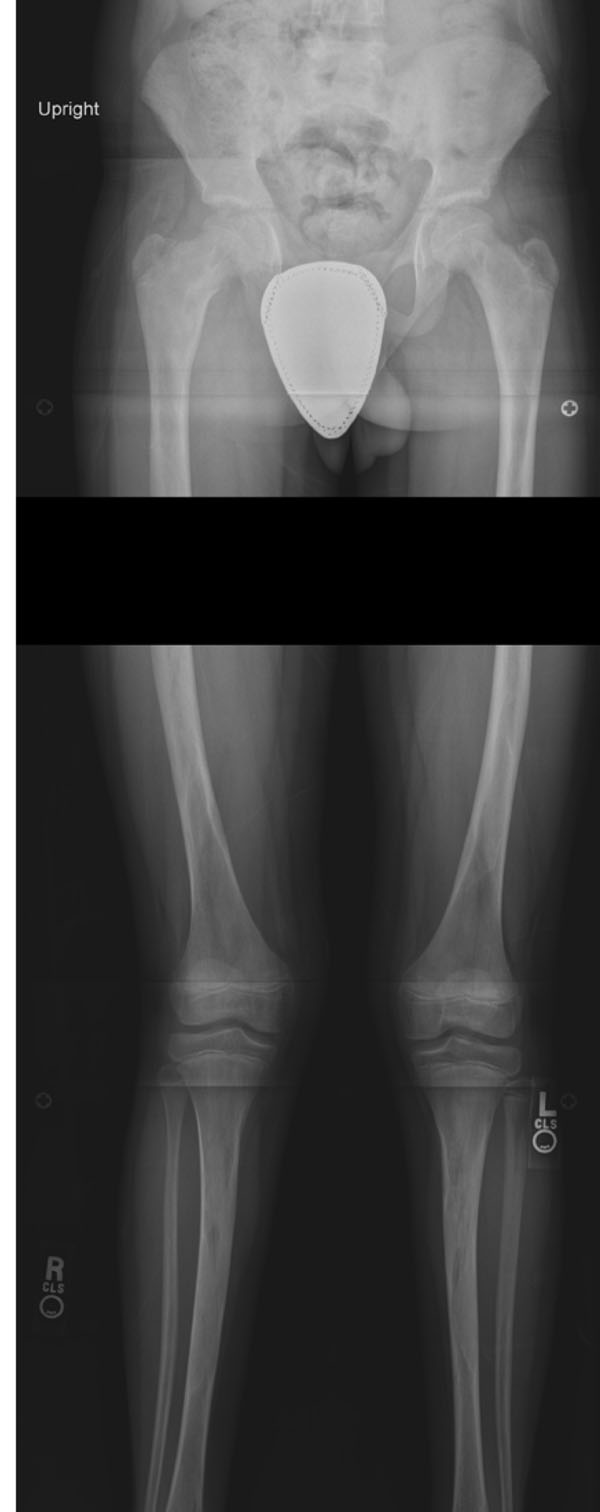
Long standing anterior/posterior (A/P) radiograph. This single view A/P film of both extremities with the patient standing allows for assessment of the extent of FD in both legs, hip angulation, and for potential limb length discrepancy. This radiograph demonstrates involvement of both femurs, both tibias, and early deformity of the upper right femur with decreasing neck-shaft angle.

Treatment of PFD in children during the growing years is often very challenging. Patients may present across a broad spectrum of clinical involvement. The initial presenting extent of bone involvement is often misleading, especially in the young child. Small areas of involvement may escape detection by the initial bone scan if the child is less than six years of age, and most areas will expand in size subsequent to the initial detection in the young child. Most patients will develop fractures and long bone deformity in the absence of surgical intervention. In the absence of a fracture or symptoms, the follow-up for a child with FD consists of twice yearly clinical evaluations with special attention to limited range of motion, obvious angular deformity and limb length discrepancy. The appendicular skeleton can often be evaluated without radiographs, with the exception of the proximal femur, where deformity may be progressive with little visible deformity until the angulation is severe (Fig. 1). Therefore, when disease is present in the proximal femur, radiographs should be obtained periodically. Limb length discrepancy can be an early sign of progressive deformity. Radiographs are used selectively to monitor the progression of lesions initially identified using the bone scan. Radiation exposure should be minimized; therefore the routine use of skeletal surveys is discouraged. Whenever available, single exposure, full-length standing radiographs of the entire lower extremities are the best way to assess for progressive disease, deformity, and limb length discrepancy (Fig. 2). Individual films of bones with suspected “insufficiency fractures” are obtained as needed. After the initial diagnostic bone scan, “follow-up” bone scans in the absence of a specific indication are not indicated.

## Scoliosis

**Figure 3:**
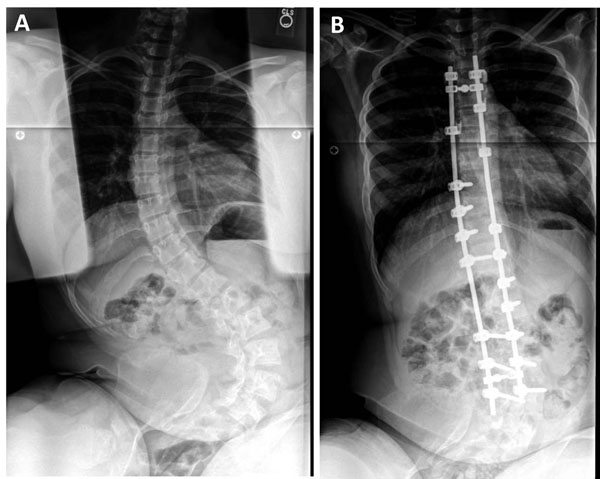
Surgical management of progressive scoliosis in a patient with fibrous dysplasia. Pre-treatment radiograph shows extensive scoliosis with both a thoracic and lumbar curve (A). The same patient is shown after posterior spinal fusion (B).

Scoliosis is common in FD and may lead to significant deformity and even rarely to death, if untreated [6,7]. In most patients it may be evaluated by clinical exam alone. However, radiographs are appropriate when the patient shows signs of increasing deformity on physical examination. Presently, there is no peer-reviewed published literature to guide the use of scoliosis bracing in FD. Bracing in typical adolescent idiopathic scoliosis modifies the alignment of the spine using indirect pressure on the spine through pressure on the ribs. As many of the patients with FD significant enough to have progressive scoliosis have rib involvement, management by bracing management is likely to be problematic and ineffecitve. For patients with significant and progressive scoliosis, surgical fusion and instrumentation is indicated (Fig. 3). Computed tomography is helpful in detecting the degree of FD in each individual vertebral segment that is to be included in the fusion. Fixation devices (hooks, screws, wires, etc.) cannot be used safely in vertebral segments with FD. Fixation should be placed in adjacent vertebral segments that are not involved in order to provide stability and correction of deformity. Standard instrumentation and fusion has been used successful in the small number of cases with which the authors have experience, and somewhat surprisingly the results of a single operation have shown excellent long-term durability.

## Fracture and deformity management

**Figure 4:**
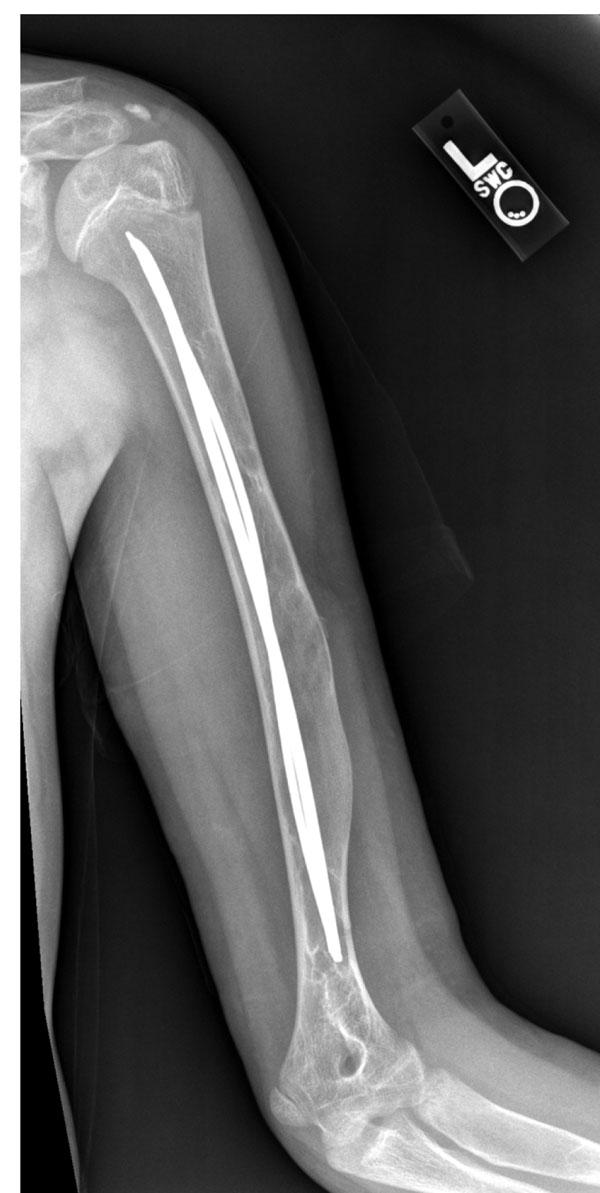
Intramedullary rods of the humerus in fibrous dysplasia. Demonstrated are flexible intramedullary rods in the humerus for the treatment of chronic upper arm pain in a patient who needed to weight bear through using their arms.

Standard closed management is often appropriate for selected upper extremity fractures. However the fractures should not be allowed to heal with residual angulation, as remodeling and correction of residual angulation does not typically occur as quickly and as reliably in FD as it would in normal bone. With that in mind, the use of internal fixation for upper extremity fractures may be considered, especially in older children. The entire child must be considered when making a decision regarding the management of upper extremity involvement. For example, in children requiring chronic use of supportive devices (i.e. crutches or canes) due to lower extremity issues, correction of deformity and internal fixation of selected upper extremity deformities is appropriate, as the upper extremities of those individuals are weight bearing (Fig. 4).

Lower extremity fractures will almost always require the use of internal fixation, although selected non-displaced tibia fractures may be managed with casts. Non-weight-bearing management should be avoided whenever possible. Patients with FD frequently have underlying bone fragility due to a combination of FD in other parts of the skeleton, metabolic issues, and diminished activity. Prolonged non-weight-bearing treatment following surgery will only aggravate the preexisting bone weakness. The use of internal fixation devices may allow early weight-bearing and should be considered when feasible. As with the upper extremity, remodeling of angulation may not occur.

**Figure 5:**
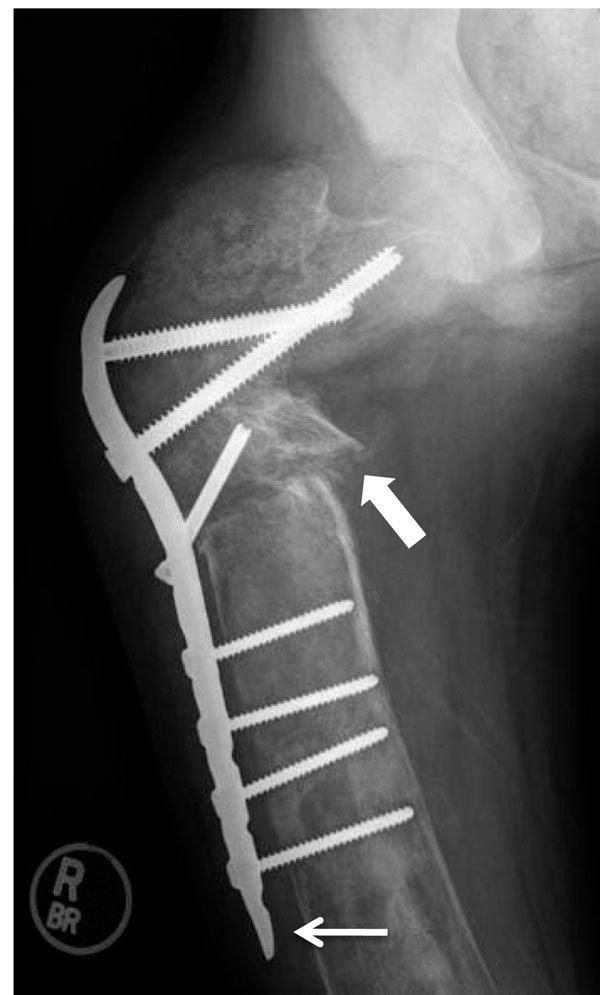
Screw and plate device in upper femur. This radiograph demonstrates the first stage reconstruction in a patient with severe deformity using a plate and screw device. The weak bone cortex results in poor fixation and eventual failure. Plate and screw constructs should be avoided if possible, or used only for temporary fixation until intramedullary fixation is possible. The thin white arrow demonstrates a problem typically encountered when fixation screws are inserted in to bone affected with FD, i.e. the plate has pulled away from the bone due to loss of screw fixation.

Ideally, deformity should be avoided, and when present corrected. The new bone formed after fractures and corrective osteotomies is dysplastic, thus recurrent fractures and deformity should be expected. In virtually all cases, the cortex of the femur and tibia is severely compromised, and therefore the use of typical plate and screw devices is discouraged, unless screws can be placed outside the FD lesions obtaining purchase in normal cortical bone. Screw failure is extremely likely if the screws are placed into FD bone and should be used with caution only in selected patients with adequate cortical bone. When screws are used, augmentation with external devices (cast or brace) may be indicated (Fig. 5). Bracing as a prophylactic treatment for deformity is ineffective. Likewise, there is no indication for prophylactic use of internal fixation devices in the absence of fracture, deformity, or chronic weight-bearing bone pain.

**Figure 6:**
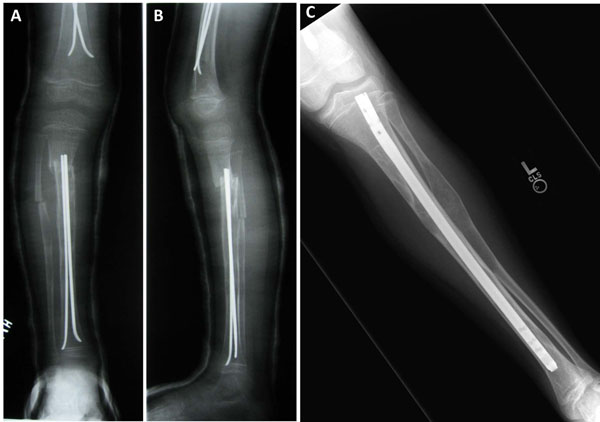
Intramedullary rods in fibrous dysplasia. The use of flexible intramedullary rods in both the femur and tibia in a small child following corrective osteotomy (A&B). A fixed intramedullary rod used in an older child is also shown (C).

The use of intramedullary (IM) devices is strongly suggested for all lower extremity fractures and reconstructions [8-10] (Fig. 6). A variety of devices are available, however, few are designed specifically to address the unique challenges of reconstruction of the proximal femur in children. The proximal femur is very commonly involved in this disease and presents the most unique reconstruction challenges. Once varus deformity occurs in the femur, realignment becomes extremely challenging. Varus below a neck-shaft angle of 130 degrees is very concerning and varus below 120 degrees may constitute an indication for surgical intervention, even in the absence of a fracture or weight-bearing bone pain [9,10]. A decline in the neck-shaft angle on sequential radiographs warrants consideration of surgical intervention. In cases where the neck-shaft angle has become severely deformed, single-staged correction may not be feasible. In selected cases, staged procedures using blade-plate or screw-plate devices to achieve partial correction may be used and later converted to IM devices when the desired correction is achieved.

**Figure 7:**
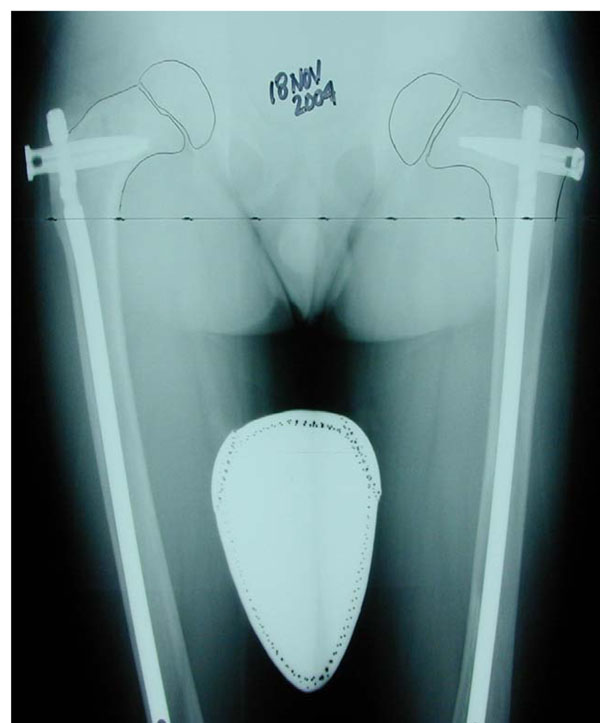
Upper extremity devices in a small femur. Demonstrated is the adaptation of rods created for use in the upper extremity in the bilateral femora of a child with FD.

Over-correction into valgus alignment in the upper femur should be considered when possible. Although this introduces a theoretical risk of abductor muscle weakness, the practical results have shown near-normal function and less frequent need for revision surgery. A study of the neck-shaft angle in children with PFD shows a correlation between normal neck-shaft angle and improved functional outcomes (5). Fixation devices designed for use in the upper extremity of adults may be adapted for use in the pediatric lower extremity on a case-by-case basis (Fig. 7). Until recently, smaller IM devices suitable for use in the upper femur were not available, however, more devices are now being manufactured and may be suitable for these reconstructions. Even when suitably sized devices are available, they are typically designed to reproduce normal childhood alignment and therefore may be difficult to use when attempting to produce a valgus alignment.

**Figure 8:**
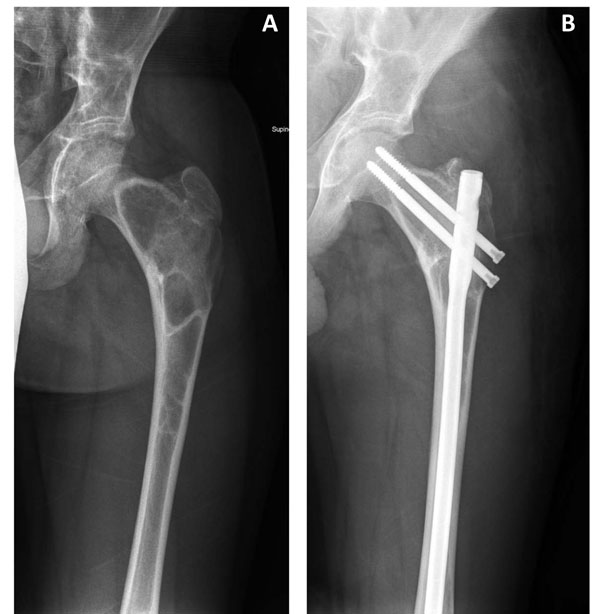
The use of a standard adult device in an adult with fibrous dysplasia. Demonstrated is the use of a standard intramedullary rod in a mature patient with fibrous dysplasia with chronic weight-bearing pain before (A) and after surgery (B).

Internal fixation devices may be used in non-deformed bone to treat frequent fractures or chronic weight-bearing bone pain (Fig.8). Fixation for bone pain should be delayed until the medical management has been optimized by the patient’s endocrinologist. The importance of proper pharmacologic management of the endocrine and metabolic aspects of this condition cannot be overemphasized, as the associated endocrinopathies (i.e. hyperthyroidism, phosphate wasting) often lead to decreased bone strength both within the FD bone and in the surrounding “unaffected” bone [11]. The use of bisphosphonates has been effective in reducing the incidence of significant weight-bearing bone pain [12,13], but has not been shown to decrease progressive deformity or to decrease the rate of fracture or surgery [14]. It is very important to counsel the parents and patients regarding the need for repeated surgical procedures to control the progressive nature of the bone deformities. This is especially problematic in young children with significant disease. As the skeleton is growing, the soft tissues exert very strong forces which will often exceed the strength of the bone that is affected with FD. Recurrent deformity will require repeated surgical procedures that become less frequent as the child reaches adult height.

Limb length discrepancy is common in PFD and is more likely to occur in patients with severe disease, requiring multiple corrective procedures. Attempts to surgically lengthen bone with FD will result in the formation of more dysplastic bone. Mechanical devices, such as circular frames with thin wire fixation, are not likely to hold in FD bone. Lengthening may be considered if there are bones or bone segments that are of good quality and not involved with FD. Epiphyseodesis of the longer limb at the appropriate time may be considered; however, many FD patients are destined to be of short stature and may not accept a procedure that reduces adult height. A patient and family that have undergone multiple major surgical procedures may prefer to accept the need to wear a permanent shoe lift as a means to deal with a limb length discrepancy, rather than accept another surgical procedure.

## Transformation of fibrous dysplasia

**Figure 9:**
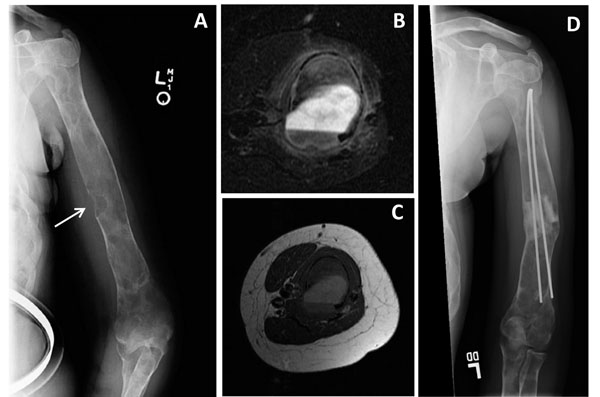
Aneurysmal bone cyst (ABC) of the upper extremity in fibrous dysplasia. This 16 year-old girl with McCune-Albright syndrome developed pain in the left humerus. A radiograph revealed a new lytic lesion that breeched the cortex (A, arrow). MRI revealed a fluid/fluid level within the lesion consistent with an ABC (B&C). The post-surgery radiograph shows a good result with the use of grafting material and flexible intramedullary rods (D).

Over time, fibrodysplastic bone may undergo transformation into either benign or malignant tumors. Transformation into aneurysmal bone cysts (ABC) may occur in any bone with FD, but has been reported most often in the skull. Aneurysmal bone cysts can also occur in many pre-existing benign bone tumors. When ABC’s form in FD bone, the already soft and dysplastic bone deteriorates into an enlarging cyst that is filled with blood. The cyst typically expands much more rapidly than FD would, leading to increasing bone pain and fracture. Unfortunately, the radiographic appearance of ABC is very similar to FD and thus is often not recognized without the use of more sophisticated studies such as MRI (Fig. 9). Surgical management is required in cases of ABC formation.

Malignant transformation in FD is very rare and most reported cases appeared to be associated with radiation therapy, which was commonly used to treat FD lesions in the past [15]. At this time, there is no indication for the use of radiation therapy in the management of FD of bone.

## Bone grafting

Bone grafting may be indicated for selected adult patients with monostotic disease [16]. Allograft is preferred to autograft to eliminate donor site morbidity. Bone grafting for patients with PFD is not useful. Attempts to completely remove polyostotic disease with curettage and bone grafting are rarely successful. Such surgery results in significant blood loss, and the FD lesions typically remodel the grafts with FD over time. There may be a limited indication for the use of allograft in conjunction with internal fixation for selected cases where the graft material provides temporary augmentation for the internal fixation. Large whole bone allographs may be used in adult patients as composite reconstructions in association with artificial joint replacement surgery in selected cases. There are always exceptions to any rule, and occasionally the small bones of the hands and fingers may suffer repeated fractures that warrants the use of grafting. These bones can often be treated effectively with curettage and bone grafting without fixation.

## Bone infection and blood loss

The majority of FD lesions are richly supplied with blood vessels, and extensive bleeding may be anticipated for patients in whom a lengthy reconstruction is planned. The presence of an ABC in the lesion can also increase the blood loss during surgery. This may become significant, especially for reconstructions where multiple corrective osteotomies are required and where the medullary canal must be reconstituted with drilling and/or reaming prior to the insertion of an IM device. Blood transfusion may be necessary if multiple sites of deformity correction are attempted at one episode of surgery. Therefore we recommend that the surgeon advocate early intervention before the development of significant bone deformity. Bone infection following surgery for FD is uncommon and perhaps less frequent than in similar surgical procedures performed in otherwise normal bone. The rich blood supply of the FD tissue may provide some degree of protection from infection in these patients. The authors have limited experience with infection in FD surgery and suggest that standard orthopedic principles of management be utilized.

## Summary

In summary, PFD is an extremely complex condition causing fractures and deformity in children. Although relatively standard procedures are effective in adults with MFD, children with PFD require aggressive and innovative intervention if severe deformity is to be avoided. Bone grafting is seldom indicated. The use of intramedullary internal fixation devices is preferred over plate and screw devices whenever possible. The management of each patient must be individualized. The expectations of the parents must be prospectively managed and the patient and parent must be prepared for multiple episodes of reconstructive surgery throughout the growing years.

## Competing interests

The authors declare that they have no competing interests.
